# Insight Into the Molecular Mechanisms for Microcystin Biodegradation in Lake Erie and Lake Taihu

**DOI:** 10.3389/fmicb.2019.02741

**Published:** 2019-12-10

**Authors:** Lauren E. Krausfeldt, Morgan M. Steffen, Robert M. McKay, George S. Bullerjahn, Gregory L. Boyer, Steven W. Wilhelm

**Affiliations:** ^1^Department of Microbiology, The University of Tennessee, Knoxville, Knoxville, TN, United States; ^2^Department of Biology, James Madison University, Harrisonburg, VA, United States; ^3^Great Lakes Institute for Environmental Research, University of Windsor, Windsor, ON, Canada; ^4^Department of Biological Sciences, Bowling Green State University, Bowling Green, OH, United States; ^5^Department of Chemistry, College of Environmental Science and Forestry, State University of New York, Syracuse, NY, United States

**Keywords:** biodegradation, cyanotoxins, cyanobacteria, harmful algal blooms, RNA-sequencing

## Abstract

Microcystins are potent hepatotoxins that are frequently detected in fresh water lakes plagued by toxic cyanobacteria. Microbial biodegradation has been referred to as the most important avenue for removal of microcystin from aquatic environments. The biochemical pathway most commonly associated with the degradation of microcystin is encoded by the *mlrABCD* (*mlr*) cassette. The ecological significance of this pathway remains unclear as no studies have examined the expression of these genes in natural environments. Six metatranscriptomes were generated from microcystin-producing *Microcystis* blooms and analyzed to assess the activity of this pathway in environmental samples. Seventy-eight samples were collected from Lake Erie, United States/Canada and Lake Tai (*Taihu*), China, and screened for the presence of *mlr* gene transcripts. Read mapping to the *mlr* cassette indicated transcripts for these genes were absent, with only 77 of the collective 3.7 billion reads mapping to any part of the *mlr* cassette. Analysis of the assembled metatranscriptomes supported this, with only distantly related sequences identified as *mlrABC-*like. These observations were made despite the presence of microcystin and over 500,000 reads mapping to the mcy cassette for microcystin production. Glutathione S-transferases and alkaline proteases have been previously hypothesized to be alternative pathways for microcystin biodegradation, and expression of these genes was detected across space and time in both lakes. While the activity of these alternative pathways needs to be experimentally confirmed, they may be individually or collectively more important than *mlr* genes in the natural environment. Importantly, the lack of *mlr* expression could indicate microcystin biodegradation was not occurring in the analyzed samples. This study raises interesting questions about the ubiquity, specificity and locality of microcystin biodegradation, and highlights the need for the characterization of relevant mechanisms in natural communities to understand the fate of microcystin in the environment and risk to public health.

## Introduction

Microcystins (MCs) are the one of the most frequently detected cyanotoxins within freshwater harmful cyanobacterial blooms (cyanoHABs). With cyanoHABS on the rise around the world, understanding the fate of MCs in the environment has assumed greater importance as they pose both ecological and public health risks. MCs can be potent protein phosphatase 1 and 2A inhibitors as well as potential tumor promoters. Consumption of MCs can result in acute hepatocytosis, cancer and various gastrointestinal problems (Carmichael, 2001; Zurawell et al., 2005; Carmichael and Boyer, 2016). Conventional municipal water treatments are effective at removing or inactivating cyanobacterial cells, but the methods risk the release of MCs from the cell. MCs are not removed by flocculation processes and require secondary treatments that include the use of activated carbon, which is effective but costly (He et al., 2016). Indeed, liver cancer and colorectal cancer has been linked to nearby bodies of water plagued with toxic cyanobacteria in the United States, Serbia and China (Carmichael, 2001; Hernández et al., 2009; Svircev et al., 2009). Cases of mortality and morbidity due to cyanotoxins have been reported since the 1800s involving birds, livestock, dogs, fish, and even humans (Chorus and Bartram, 1999; Wood, 2016). The number of cases has risen gradually over time and it is predicted that the number of individuals affected is greatly underestimated (Wood, 2016).

Microcystins are chemically stable and resistant to many abiotic factors, which has led to numerous studies questioning their fate in the environment (Harada and Tsuji, 1998). Whereas their disappearance has been attributed to some abiotic effects including dilution, adsorption, and photodegradation (Harada and Tsuji, 1998; Corbel et al., 2014), they are generally considered to be resistant to abiotic factors. MCs are also resistant to the activity of common peptidases (Tsuji et al., 1994; Mazur and Plinski, 2001; Smith et al., 2010) due to their cyclic structure and the alternating incorporation of the non-protein “R” stereoisomers of the amino acid, coupled with “iso” peptide bonds formed through the side chains of glutamate and aspartate. Despite these challenges, microorganisms that span multiple phyla and even domains are reported to degrade MCs (Supplementary Table S1). Further, experiments assessing the biodegradation of MCs which employ natural microbial communities from lakes (Christoffersen et al., 2002; Edwards et al., 2008; Eleuterio and Batista, 2010; Mou et al., 2013; Dziga et al., 2017; Lezcano et al., 2018), drinking water reservoirs (Cousins et al., 1996; Ho et al., 2012), estuary and sea water (Lemes et al., 2008), water treatment facilities (Lam et al., 1995; Saito et al., 2003b; Ma et al., 2014), soil (Miller and Fallowfield, 2001; Bibo et al., 2008; Cao et al., 2018; Redouane et al., 2019), lake sediments (Rapala et al., 1994; Cousins et al., 1996; Chen X. et al., 2010; Song et al., 2014; Li et al., 2016; Zhu et al., 2019) and biofilters (Grützmacher et al., 2002; Ho et al., 2006; Lee et al., 2006; Ho et al., 2007; Hoefel et al., 2009; Ho et al., 2012; Kumar et al., 2019) have demonstrated that biodegradation capacity is seemingly ubiquitous across a wide range of environments and occurs under a variety of conditions (Li et al., 2017). For these reasons, the current literature often refers to biodegradation as the most important route for the disappearance of MCs in nature (Christoffersen et al., 2002; Holst et al., 2003; Chen et al., 2008; Grützmacher et al., 2009; Bukowska et al., 2018).

The reported genetic pathway involved in MC biodegradation involves four enzymes encoded by *mlrA*, *mlrB*, *mlrC*, and *mlrD* (Bourne et al., 1996; Bourne et al., 2001). This pathway has been primarily associated with the biodegradation of MC-LR, a prevalent congener in nature (Carmichael and Boyer, 2016). MlrA, termed microcystinase, is a metalloprotease linearizing the MC-LR at the Arg-ADDA bond. It is commonly used as marker gene and considered to catalyze the most important step in biodegradation of MCs (Dziga et al., 2012; Dexter et al., 2018; Lezcano et al., 2018). MlrB is a serine protease that drives the hydrolysis of the linearized MC-LR at the Ala-Leu bond, forming a tetrapeptide. The tetrapeptide is then subject to further degradation by a second metalloprotease, MlrC, and it was demonstrated that MlrC can also cleave the linearized MC at the -Adda group (Dziga et al., 2012). MlrD is a putative transporter, but whether MC biodegradation occurs intracellularly or extracellularly remains inconclusive (Bourne et al., 2006).

This pathway has been extensively studied in culture (Supplementary Table S1, Bourne et al., 2006; Dziga et al., 2012; Maseda et al., 2012; Dziga et al., 2016; Dexter et al., 2018; as examples), although not all MC degrading isolates have tested positive for these genes. Further, the environmental relevance and distribution of the *mlr* cassette in the environment remains unclear. The *mlrA* gene has been detected in DNA isolated from microbial communities with MC degradation capabilities from freshwater samples (Mou et al., 2013; Kansole and Lin, 2016; Lezcano et al., 2018), but more commonly environments where MC biodegradation has been tested are not screened for use of this pathway. More often, the *mlrA* gene has been detected in enriched samples such as biofilms, constructed wetlands, filtration sand and water samples from treatment plants (Grützmacher et al., 2002; Bourne et al., 2006; Ho et al., 2006; Hoefel et al., 2009; Ho et al., 2012). Other genes in the cassette are not as widely studied in the environment and sequences of proteins with confirmed function are lacking, particularly for *mlrC*. By use of the same PCR primers (Saito et al., 2003a) and narrowly targeted degenerate qPCR primers (Hoefel et al., 2009) used amongst many studies, the understanding of the diversity of this pathway remains uncertain and makes a negative PCR result difficult to interpret. Further, since the methods used to screen for *mlrA* in native microbial communities have been based on DNA sequences (Hoefel et al., 2009; Mou et al., 2013; Zhu et al., 2014; Lezcano et al., 2018), only the potential of the pathway to be a route for MC biodegradation has thus far been considered. The activity of all the genes in the *mlr* pathway, either in the form of transcribed RNA or expressed proteins, has not been measured in natural samples.

Our goal in this study was to survey the natural environment for evidence of microcystin biodegradation during toxic blooms by examining the relative expression of the genes in the *mlr* pathway. We also screened for transcripts of other hypothesized mechanisms for MC biodegradation, including glutathione S-transferases (GSTs), CAAX proteases and alkaline proteases (Takenaka and Watanabe, 1997; Mou et al., 2013). Knowledge of MC biodegradation in the natural environment on a molecular level has potential use in predicting the capacity of the native microbial community to remove MCs from that environment and identifying environmentally relevant mechanisms that are responsible. This was done using shotgun metatranscriptomes from six surveys in eutrophic freshwater lakes with a combined total of 3.7 billion reads. The use of metatranscriptomes relative to DNA-based assays allows for a more accurate representation of the active function of the microbial community. Metatranscriptomes can also provide insight on the proportional representation of a gene’s activity within a community and provide the ability to detect more divergent sequences of the genes of interest.

## Materials and Methods

### Sample Collection and Sequencing

**FIGURE 1:**
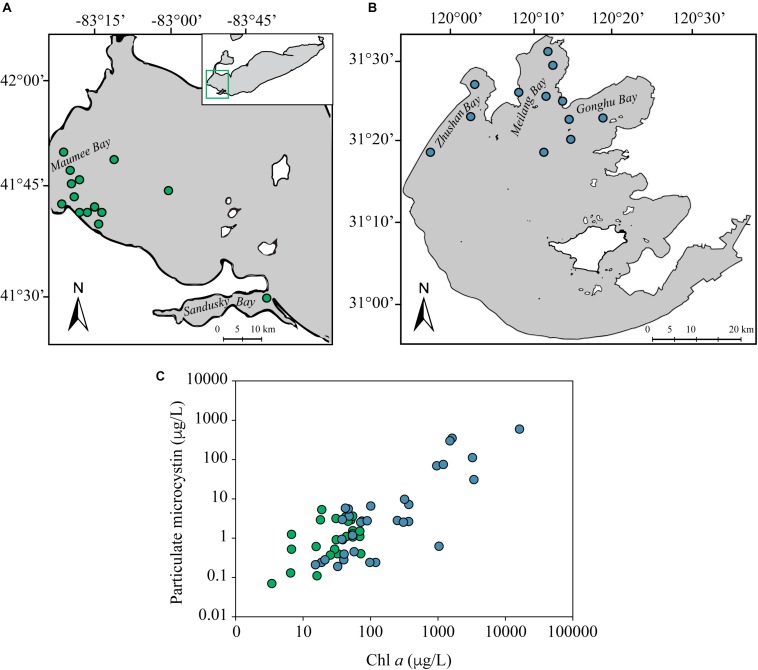
Map of Lake Erie **(A)** and *Taihu* **(B)**. Stations sampled for metatranscriptomes are depicted as green (Lake Erie) or blue (*Taihu*) circles. Coordinates for each sample location can be found in Supplementary Tables S3, S4. Particulate microcystin and chl *a* dynamics depict the range of bloom severity and toxicity amongst samples used in the metatranscriptomic analysis **(C)**. The particulate microcystin and chl *a* concentrations for each sample can be found in Supplementary Tables S3, S4.

A total of six surveys, encompassing seventy-eight metatranscriptomes with a wide range of environmental conditions and MC concentrations, were used to screen for the expression of genes involved in MC biodegradation (Figure 1). Thirty-seven of the metatranscriptomes were generated from surface waters in the western basin of Lake Erie (United States/Canada) with sixteen from a bloom event in early August 2014 across eight stations (Steffen et al., 2017), seven tracking a bloom over a diurnal cycle in late August 2014 (Davenport et al., 2019), five from a bloom event in July 2013 from two stations (Steffen et al., 2017) and nine from three stations in August 2012 (Steffen et al., 2015). The Lake Erie dataset from early August of 2014 by Steffen et al. (2017) was of special interest because samples were taken at and around the City of Toledo’s water intake where dissolved MC concentrations in the source water were above 10 μg/L and exceeded 2.5 μg/L in the finished water, resulting in a 3 day water ban affecting 400,000 people (Carmichael and Boyer, 2016; Steffen et al., 2017).

Forty-one metatranscriptomes were generated from surface water collected in the northern part of Lake Tai (*Taihu*), China in 2013 and 2014 during bloom events. The *Taihu* 2014 metatranscriptomes were composed of thirty-five samples collected across nine locations at monthly intervals from June to October, capturing an entire bloom event (Stough et al., 2017; Tang et al., 2018). Six metatranscriptomes were generated from samples collected in August 2013 from six stations. Sample collection, RNA extraction and sequencing for the *Taihu* 2013 metatranscriptomes were performed using the same approaches as previously described for published studies on Lake Erie (Steffen et al., 2015). The *Taihu* 2013 metatranscriptomes are publicly available on the MG-RAST (Meyer et al., 2008) server (ID: mgm4768721, mgm4768720, mgm4768724, mgm4768726, mgm4768728, mgm47687130) and the Lake Erie 2014 diel metatranscriptomes are available from the NCBI SRA database (SRP128942, SRP128954, SRP128945, SRP117911, SRP117914, SRA117922, SRA117915). More information on data availability, location, dates of sampling and environmental parameters for all metatranscriptomes can be found in Supplementary Tables S2–S4 or in the citations above.

### Read Recruitment to the *mlr* Cassette

Expression of *mlr* pathway was first evaluated by recruiting reads from Lake Erie and *Taihu* metatranscriptomes to the *mlrA-D* from *Sphingomonas* sp. ACM3962 in CLC Genomics Workbench (Qiagen, Hilden, Germany). Reads were trimmed with a quality limit of 0.05 and an allowance of up to two ambiguous base pairs using CLC Genomics Workbench. Recruitments were performed using only reads >100 bp for all metatranscriptomes and a similarity fraction of 0.8 and a length fraction of 0.5 to encompass known and potential unknown diversity of these genes. Since the 2012 Lake Erie dataset generated reads of only 50 bp, slightly more stringent parameters were used to reduce non-specific mapping (length fraction 0.97 and similarity fraction 0.8). Reads were also recruited using the same metrics to *mlrA-D* from *Sphingopyxis* sp. strain C-1, *Rhizobium* sp. TH, and *Novosphingobium* sp. THN1. For comparisons, these reads were recruited to the *mcy* operon (*mcyA-J*) from the model species *Microcystis aeruginosa* NIES 843 with the more stringent similarity fraction and length fraction of 0.9 that still allowed for the inclusion of other toxin producing species (Steffen et al., 2017).

### Screening for *mlrABC*, GSTs and Alkaline Proteases in Assembled Data

Reads were assembled from individual samples into contiguous sequences (“contigs”) in CLC Genomics Workbench using default parameters (bubble size = 50). All contigs >200 bp from the metatranscriptomes were screened using BLASTx against a locally curated database containing the currently available MlrA, MlrB, and MlrC amino acid sequences in NCBI’s GenBank. Contigs with positive hits to MlrA, MlrB, and MlrC (contigs with an *E*-value of <0.00001) were collected and translated to amino acid sequences using the appropriate reading frame identified by BLASTx. To compare active sites, sequences for MlrA, MlrB, and MlrC were aligned to their respective candidate contigs in CLC Genomics Workbench. This method of *mlr* detection was also applied to a metagenome from a study on Lake Erie where MC biodegradation and *mlrA*-like genes have previously been reported (Mou et al., 2013) as well as a metatranscriptome generated from samples from a winter diatom bloom (Edgar et al., 2016). All contigs detected using these methods can be found in the Supplementary Data Sheet S1.

Due to the phylogenetic diversity of GSTs and alkaline proteases, these sequences were identified in the metatranscriptomes by first uploading all assembled data to MGRAST. Contigs were annotated using the best representative hit and an *E*-value of <0.00001 against the SEED database for functional classification and Refseq for taxonomic assignment. It should be noted that no contigs were annotated as *mlr* genes. Contigs were first filtered by phyla, only including those from the phyla Proteobacteria, Actinobacteria, Bacteroidetes, and Firmicutes. These phyla were chosen because in most of the metatranscriptomes, these were classified as the most highly active non-cyanobacterial prokaryotic transcripts. In addition, all currently known MC degrading bacteria are within these phylogenetic groups. Contigs or portions of contigs annotated as GSTs (E.C. 2.5.1.18) filtered by representative hit were downloaded and reads were mapped with high stringency (0.97 similarity fraction and length fraction) and normalized by contig length and library size to determine expression of these genes in each sample. This stringent parameter was used so as to only recruit very specific reads to each contig. The only annotated alkaline protease contigs present in any metatranscriptomes from the phyla of interest were annotated as secreted alkaline metalloproteinase PrtA/B/C/G (E.C. 3.3.24.-). Expression of these genes were determined as previously described for GSTs. Shadeplots were constructed in GraphPad Prism v7.03 after being log_10_ transformed for visualization, and the shading of each box represents levels of total expression in a sample from a particular gene; the darker the shading (the more positive the number) indicates higher expression. All assembled data can be found on MGRAST and sample IDs are available in Supplementary Tables S3, S4.

### Phylogenetic Analysis of MlrA, MlrB and MlrC Candidate Contigs

Amino acid reference sequences for CAAX protease and bacteriocin processing (CPBP) intramembrane metalloprotease family proteins were downloaded from UNIPROT as well as the top BLASTx results from the NCBI Refseq database for each *mlrA*-like contig were used to build a reference tree for phylogenetic analysis of MlrA candidate contigs. The 100 most closely related sequences in the Refseq database to MlrB and MlrC according to BLASTx were used as reference sequences for MlrB and MlrC candidate contigs. Bacterial beta-lactamase amino acid sequences from UNIPROT with confirmed function were also incorporated into MlrB reference tree. Alignments of reference sequences were performed using MUSCLE in Mega v7.0 with eight iterations (Tamura et al., 2007). Maximum likelihood trees were made using the PhyML server according to the LG model, and likelihood ratios and branch support were calculated using a Shimodaira-Hasegawa (SH)-like approximate likelihood ratio test (aLRT-SH-like, Guindon et al., 2010). Contigs were incorporated into the tree using pplacer^1^ as in Krausfeldt et al. (2017).

## Results

### Sample Assessment

Measured particulate concentrations of MC verified that the majority of the samples were toxic and in conjunction with chl *a* concentrations indicated these samples ranged in bloom severity and toxicity levels (Figure 1C and Supplementary Tables S3, S4). Measurements of dissolved MC, particulate MC and chl *a* from 2015–2017 in Lake Erie indicated the presence of particulate MC and chl *a* corresponded to the presence of dissolved MC throughout blooms events (Supplementary Figure S1).

### Read Mapping to the *mlr* Cassette

**FIGURE 2:**
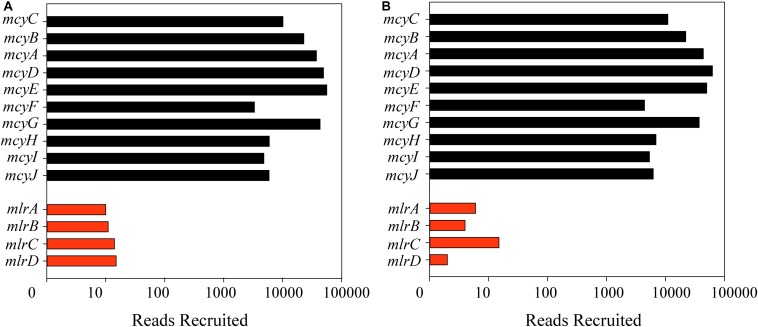
Number of reads mapped on a log scale from metatranscriptomes from Lake Erie **(A)** and *Taihu* **(B)** to the *mlr* cassette (red bars) for *Sphingomonas* ACM3962 for microcystin biodegradation (at a similarity fraction of 0.8 and length fraction of 0.5) and *mcy* cassette (black bars) from *Microcystis aeruginosa* NIES843 for microcystin production (at a similarity and length fraction of 0.9).

Reads from each metatranscriptome were first recruited to all four genes in the *mlr* pathway from *Sphingomonas* sp. ACM3962 with parameters set to account for diversity of known *mlr* sequences (Supplementary Figure S2). Only 77 reads out of the total of over 3.5 billion reads surveyed mapped to genes in the *mlr* pathway; 50 from Lake Erie, and 27 from *Taihu* (Figures 2A,B). Notably, mapped reads did not provide full coverage of any of the individual genes in the *mlrABCD* cassette. Reads were also mapped to the *mlr* cassette identified in other MC degraders, and similarly, few reads mapped with no full coverage of any gene in the cassette (Supplementary Table S5). Using the same parameters for the *mcy* operon, over 3,000,000 reads mapped to the *mcy* genes. When using more stringent parameters, which increased the similarity necessary for mapping, almost 500,000 reads mapped to the *mcy* operon, the genes for MC production (Figures 2A,B).

### Screening for *mlrA*, *mlrB*, and *mlrC*

**FIGURE 3:**
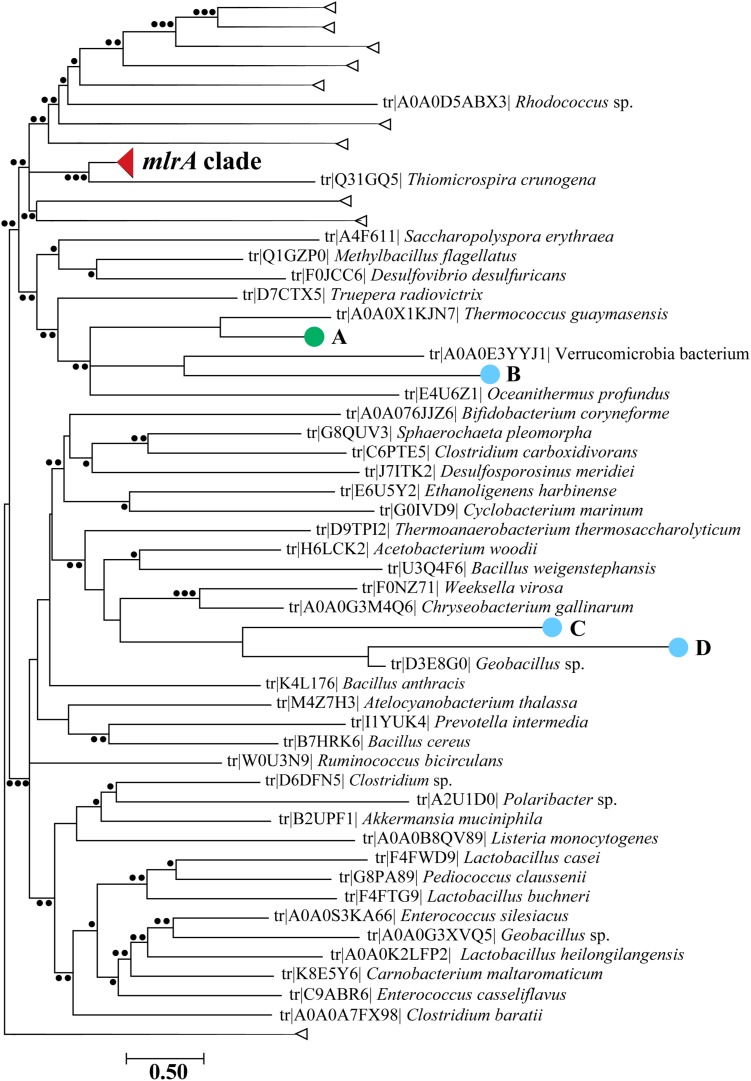
Maximum likelihood phylogenetic tree with candidate *mlrA* sequences aligned using pplacer indicated by large closed circles (light blue = Lake Erie Diel 2014; green = Lake Taihu 2013). The letters correspond to which sample they were found: **A:** *Taihu* 2013 at the dock; **B:** Lake Erie (Diel) 2014 1600 h; **C:** Lake Erie (Diel) 2014 2200 h; **D:** Lake Erie (Diel) 2014 2200 h. The *mlrA* clade is collapsed for visualization purposes in a closed red wedge and other collapsed branches are denoted by open wedges. Smaller closed circles at nodes represent likelihood ratios. ∙∙∙ > 90, ∙∙ > 70, ∙ > 50.

Reads from each metatranscriptome were assembled into contigs and screened for the marker gene *mlrA* using BLASTx. Only ten contigs were identified by this analysis to be *mlrA-*like. After screening these contigs against the Refseq database using BLASTx, four contigs were annotated as part of the same protein family (CPBP) as *mlrA* (Pei et al., 2011) due to the presence of an *abi* protein domain, and these were considered potential *mlrA* candidate transcripts. Three contigs were from the 2014 diel study on Lake Erie and one was from *Taihu* in 2013. BLASTx indicated all sequences shared ∼37 to ∼57% identity and ∼54 to ∼84% positives with at least one MlrA sequence with known function, but the *E*-values were all >1 × 10^–14^ (Supplementary Data Sheet S1). Further phylogenetic analysis using translated protein sequences was performed, and all of these contigs were very distantly related to sequences within the MlrA clade (Figure 3). Over 200 contigs were identified as *mlrB* candidates while only nine were identified as *mlrC* candidates (Supplementary Figure S3). Phylogenetic analysis indicated there were seven clades of MlrB-like contigs, and these contigs were detected in both lakes. Both MlrB and MlrC candidate sequences were distantly related to the tightly clustered clades of MlrB and MlrC sequences with confirmed function. Alignments of reference MlrA and MlrC sequences with respective contigs indicated the MlrA and MlrC candidate transcripts did not contain the appropriate active site residues needed for functionality against the MC molecule or target degradation products (Supplementary Figures S4, S5), although some of the contigs were not long enough to be aligned to that region. Many MlrB candidate sequences did contain the residues within the active site needed to function properly against linearized MC (Supplementary Figure S6).

For comparison, this technique was also used on 454-generated metagenomic data from Mou et al. (2013) from mesocosm experiments using water from Lake Erie to screen for *mlrA*. Four metagenomes were generated: two that followed treatment with MCs and two which were controls to examine changes in community structure and function upon MC additions. One 500 bp sequence from one MC treatment shared 95.63% identity with *mlrA* from *Sphingopyxis* sp. C1 (a sequence used for screening in the analysis above) with an *E*-value of 0, demonstrating the ability of the technique used here to identify genes of interest. Other sequences that shared identity with *mlrA* had an *E*-value > 0.00001, indicating there was a very low probability that these shared the same function as *mlrA*. Analysis of a metatranscriptome from a non-cyanobacterial bloom event in the winter yielded no *mlrA* or *mlrA-*like sequences.

### Determining Expression of *mlrA*-Like Genes and Genes in Alternative Pathways

**FIGURE 4:**
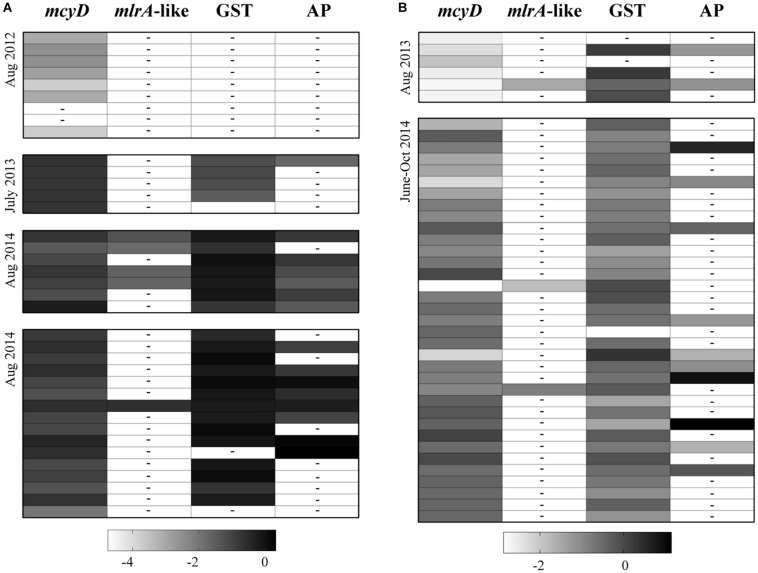
Shadeplots representing a qualitative comparison of reads that mapped to candidate *mlrA* sequences (*mlrA*-like), GSTs and alkaline protease (AP) contigs from non-cyanobacterial prokaryotes, and *mcyD* from *M. aeruginosa* NIES843, across all samples collected from Lake Erie **(A)** and Lake Taihu **(B)**. The reads that mapped to any putative genes of interest were totaled within each sample and normalized by contig length and total library size. Shading in each box represents total expression levels for each gene; the darker the color (more positive the number) the higher the expression. For visualization purposes, these numbers were log transformed. A dashed line within a box depicts a value of 0. Library sizes and values used for the shadeplot prior to transformation can be found in Supplementary Tables S3, S4. Samples in the shadeplot also appear in the same order as they do in the tables for reference.

To determine if the putative CPBP genes (*mlrA*-like genes), identified by BLASTx were potential candidates for MC removal, reads from the samples in which the contigs were found were mapped to these respective contigs. Reads mapped accounted for a low percentage of the total number of reads in each individual sample, with only 72 reads mapping in total (Figure 4 and Supplementary Tables S3, S4). Annotations assigned from the SEED database were used to try to identify more contigs that were transcripts for proteins with the CPBP family. All of them were annotated as abortive infection proteins and of cyanobacterial origin, so they were excluded from further analysis.

Contigs were identified across all studies in Lake Erie and *Taihu* as putative GSTs and putative alkaline proteases, with the exception of Lake Erie in 2012 (Figure 4). Reads from samples with putative GSTs or alkaline proteases were mapped to the respective contigs to compare relative abundances of reads from these genes to *mlrA*-like genes (Figure 4 and Supplementary Tables S3, S4). Expression of *mlrA*-like genes was generally lower than GSTs or alkaline proteases in samples where *mlrA*-like genes were detectable. Trends in temporal and spatial distribution of GST and alkaline protease transcripts were more similar to those observed for *mcyD* transcripts.

## Discussion

With the prevalence and increased severity of cyanoHABs worldwide, it is important to understand the fate of the toxins produced during these blooms. The capacity for MC biodegradation has been demonstrated in microbial communities from a variety of environments all over the world suggesting biodegradation of MCs is ubiquitous. The *mlr* pathway is currently the only characterized mechanism for MC biodegradation, however, there are many gaps regarding the diversity and ubiquity of this pathway especially in the natural environment. Many studies only use PCR approaches to determine if *mlr* is present in either isolates, sand filters or environmental samples (as examples, Chen J. et al., 2010; Yang et al., 2014a; Lezcano et al., 2016; Dziga et al., 2017; Thees et al., 2018). One limitation to PCR-based approaches for detecting *mlr* genes is that more diverse sequences may be missed due to mismatch of primers. Currently, all of the publicly available *mlrA* sequences share 87–100 percent identity on both the nucleotide and amino acid level (Supplementary Figure S2). Repeated use of the primers based on highly identical sequences may explain why there are still few representative *mlrA* sequences available outside of Alphaproteobacterial lineage and consequently, could explain why many MC degraders remain “negative” for a microcystinase homolog. Even degenerate primers that can capture more diverse sequences can be difficult to construct when reference sequences are highly identical. Genome sequencing of MC degraders, shotgun metagenomes and metatranscriptomes provide the ability to expand the knowledge about the diversity of genes not yet discovered, but few studies have employed these mechanisms to study MC biodegradation specifically (Mou et al., 2013; Maghsoudi et al., 2016; Wang et al., 2018; Zhang et al., 2018).

In this study, 78 metatranscriptomes encompassing both daily and seasonal bloom dynamics across several years were analyzed to investigate mechanisms of MC biodegradation during toxic cyanobacterial blooms. Despite the notion that MC biodegradation is ubiquitous across different environmental samples worldwide and the presence of active toxin-producing cyanobacterial species in each sample from Lake Erie and *Taihu*, there was little evidence to suggest the *mlr* pathway was an active mechanism for MC removal from these lakes. Very few reads mapped to the *mlr* cassette from four MC degrading bacteria and read mapping did not provide full coverage across any of the genes to indicate transcripts were present. These results suggested that none of the genes in the *mlr* pathway were expressed concomitantly or alone in these samples. This was supported by phylogenetic analysis of MlrA candidate sequences identified by BLASTx: all were distantly related to MlrA suggesting transcripts for this marker gene for MC biodegradation was absent. However, these sequences were part of the same protein family as MlrA, the CPBP family. While the function of these proteins in prokaryotes remains largely unclear, they are typically annotated as CAAX proteases or abortive infection proteins, and the literature suggests they may confer immunity to antimicrobial peptides secreted by the self or other bacteria (Pei et al., 2011; Barrett et al., 2012). A role for these proteins in MC biodegradation has been hypothesized previously (Kansole and Lin, 2016), but this was based on presence/absence and remains to be experimentally confirmed. The analyses presented here suggested CAAX protease transcripts were not prevalent across or within samples, and the transcripts detectable were not highly expressed and did not contain the appropriate active site for functionality (Dziga et al., 2012).

In agreement with the read recruitments, BLASTx screening confirmed that MlrB and MlrC were likely not being expressed in any of the metatranscriptomes either. All MlrB and MlrC candidate transcripts were distantly related to MlrB and MlrC sequences with known function against MCs. Similarly to MlrA candidates, few MlrC candidate sequences were identified. The diversity and prevalence of MlrB candidate sequences is interesting as MlrB has been called a silent member of the *mlr* gene cluster in some MC degraders (Jiang et al., 2011), and yet also a better biomarker for MC biodegradation than *mlrA* (Tsao et al., 2017). Many of the MlrB candidates contained the residues within the active site required to be functional, but this active site is conserved within the Beta-Lactamase protein family, and may not be a good predictor of function against linearized MC. Therefore, phylogenetic analyses alone were not sufficient to conclude these were indeed distantly related proteins with the same function. Still, this may have suggested there more diverse *mlr* genes that would not be captured by currently published primer sets or that there are more promiscuous enzymes in the environment with the ability to degrade MC. Ultimately these data suggest that transcripts from the *mlr* pathway were absent.

Microcystin biodegradation has been observed by the natural microbial community residing in Lake Erie (Mou et al., 2013) and *Taihu* (Chen et al., 2008; Li et al., 2016) previously, and several bacterial strains reported to degrade MCs have been isolated from both lakes as well (Hu et al., 2009; Chen J. et al., 2010; Jiang et al., 2011; Yang et al., 2014a, b; Zhu et al., 2016; Krishnan et al., 2018; Thees et al., 2018). The potential for MC biodegradation *via* the *mlr* pathway was also evident in both lakes; several *mlrA*^+^ MC degraders have been isolated from samples collected from *Taihu* (Supplementary Table S1), and the present study confirmed *mlrA* was detectable in the metagenomes from Lake Erie. Interestingly, only one *mlrA*^+^ isolate has been collected from the water column in *Taihu* (Jiang et al., 2011) while several others were isolated from sludge and sediment (Yang et al., 2018a, b; Supplementary Table S1). Indeed, MC biodegradation by the *mlr* pathway might be more prevalent in lake sediments (Wu et al., 2015; Bukowska et al., 2018) which were not specifically examined here. This is potentially supported by previous observations in Lake Erie: MC degrading bacteria cultured were all *mlrA*^–^ and isolated from surface water or a visible cyanobacterial bloom. It is possible that *mlr* harboring microorganisms more commonly occupy niches besides the water column, like the sediments, in which case the timing and location of MC biodegradation by the *mlr* pathway in the water column will be highly dependent on lake geomorphology and climatic events. This may explain why no *mlr* transcripts were detectable in the metatranscriptomes in the current study despite the presence of MC; they were all generated from water or scum samples collected at the surface.

Assuming the genetic potential for the *mlr* pathway did exist in the water column, the absence of an active *mlr* pathway could to due to a number of factors. It is possible *mlr*^+^ microbes existed in low abundance during the blooms due to competition. This was proposed in Lezcano et al. (2018) where increased abundances of *mlrA* gene copies were observed during bloom decline in the San Juan Reservoir in Spain, and the work by Zhu et al. (2014) on Lake Dianchi in China generally supported these observations. However, since the general activity (by proxy of transcripts) of other non-cyanobacterial prokaryotes is readily detectable even during dense blooms (Steffen et al., 2012; Chen et al., 2018), this suggested that competition with cyanobacteria was not solely responsible for the low abundance of *mlr*^+^ microorganisms or absence of *mlr* gene expression. Although the sample collection encompassed a wide range of environmental conditions (temperature, pH, nutrient concentrations, and oxygen levels), rates of MC biodegradation and the effect of environmental factors vary across different habitats (Li et al., 2017). Activity of the *mlr* pathway could have required specific conditions not captured in this study. However, rates of biodegradation are usually higher in lakes with bloom history (Li et al., 2017), which may mean transcripts were actually fleeting, and they were missed in this analysis. It is also possible these genes were transcribed at such a low level that they were not detectable or that they were simply missed during the sampling time despite the spatial and temporal sampling coverage of areas that experience blooms in these lakes.

Microcystin does induce *mlr* gene expression in culture (Shimizu et al., 2011), and many studies have enriched for MC degraders by one or more additions of MCs (i.e., Krishnan et al., 2018; Thees et al., 2018), suggesting activity of the *mlr* pathway could have been substrate limited. It is reasonable to assume that MC concentrations in the water column would be higher during bloom collapse, as observed in Lezcano et al. (2018), since MC remains intracellular (in the particulate phase) until released after cell lysis (into the dissolved phase). This could increase the abundance of *mlr*^+^ microbes and expression or *mlr* genes. However, dissolved MCs are not only present during bloom collapse; particulate MC in Lake Erie corresponded to the presence of dissolved MC (Supplementary Figure S1), and several studies that have detected dissolved MC in the water column during blooms in *Taihu* (Song et al., 2007; Su et al., 2018). This is likely due to cell turnover or lytic phage infection, which is evident in many of the samples analyzed here (Steffen et al., 2015; Steffen et al., 2017; Stough et al., 2017) and corresponded with very high particulate MC levels (several samples reaching >100 μg/L in *Taihu*). This highlights that MC was not only an available substrate at the very end of a bloom season in Lake Erie and *Taihu*. If the activity of the *mlr* pathway is dependent on higher concentrations of MC, it may not be a dominant pathway for MC biodegradation under the most environmentally relevant conditions in these lakes. Indeed, meta-analyses capturing “non-bloom” events or community responses before, during and after a bloom collapse would be useful to address the potential seasonal relevance of the *mlr* pathway in Lake Erie and *Taihu*. Currently, there are very few or no “late bloom or no bloom” metatranscriptomes to date, nor are there any metatranscriptomes generated from samples where MC biodegradation activity has been confirmed. These types community analysis will be essential in comprehensively determining the relevance of the *mlr* pathway in Lake Erie and *Taihu*.

Alternatively, the existence of other pathways for MC biodegradation has been suggested numerous times in the literature when *mlr* genes have not been detectable. No other mechanisms have been fully elucidated to date, but a few have been hypothesized. GSTs have been implicated in the detoxification of MCs in some higher plants, invertebrates and vertebrates by first conjugating to the MCs to be targeted for removal by further processes (Pflugmacher et al., 1998; Campos and Vasconcelos, 2010; Antas et al., 2018). While the role of GSTs in bacterial degradation of MCs has not been confirmed, they can play a role in biodegradation of other toxic compounds (Allocati et al., 2009). Further, Mou et al. (2013) observed higher abundances of GSTs where MCs were degraded in Lake Erie as well as other genes involved in xenobiotic metabolism. Takenaka and Watanabe (1997) provided experimental evidence for the role of alkaline proteases from *Pseudomonas aeruginosa* in the biodegradation of MCs, though the specific alkaline protease was not well described. With the exception of Lake Erie 2012, GSTs and alkaline proteases were highly expressed in samples collected from Lake Erie and *Taihu* compared to *mlrA*-like sequences in this study. These genes are inherently multifunctional and linking them to this process would require experimental confirmation. However, if their activity against MC is confirmed, the prevalent expression of these genes across time and space would suggest these pathways may be a more important mechanism of MC removal than the *mlr* pathway. The samples collected for the Lake Erie 2012 study were collected on 20 μm mesh and may indicate microbes expressing these genes are more prevalent in the free-living community rather than within cyanobacterial colonies or large particles. Transcripts for serine proteases (E.C. 3.4.21-) were sporadically observed as well (data not shown), demonstrated to be relevant in MC degrading Lactobacilli (Nybom et al., 2012). This is a broad categorization for enzymes that include common peptidases like trypsin, proteinase K, chymotrypsin and pepsin, and previous studies have shown that these enzymes in isolation can degrade MCs (Kankaanpää et al., 2005). Others have noted this would be a slow process compared to other peptides and proteins (Smith et al., 2010). Collectively, the observations in this study raise interesting questions about the specificity, diversity and ubiquity of MC biodegradation pathways in the environment.

The characterization of both *mlr* and non-*mlr* mechanisms for MC biodegradation in the natural environment is needed, as well as further investigation into the locality, abundance and activity of native MC degrading microorganisms during blooms to understand of the fate of cyanotoxins like MCs and the risk to human health. In the present study, an extensive and quantitative survey of six studies yielding 78 metatranscriptomes from two eutrophic freshwater lakes suggested that *mlr* genes were not being expressed in either of these systems. The samples were taken during MC-producing bloom events where cyanobacteria were actively transcribing *mcy* genes for MC production and MC was measurable throughout, yet *mlr* transcripts were not detectable. Despite the detection of a small number of potential *mlrA*-like genes sequences, these do not appear to be highly expressed or widely distributed across samples examined in this multi-survey study. Primarily, these samples were collected from *Microcystis* blooms, but samples collected at station 1163 in Sandusky Bay of Lake Erie suggest this pathway was not active during *Planktothrix* blooms either (Hampel et al., 2019). This is in accordance with previous work, especially from Lake Erie, and supports the on-going hypothesis that other pathways are involved in this process. While there is a need for experimental confirmation of activity against MCs, expression of GSTs and evidence for other protease activity warrants further investigation of multiple pathways for MC biodegradation in environmental samples. The complete lack of *mlr* detection suggested either that other pathways for MC biodegradation are more active during blooms or there are specific niches where this process occurs that may not include the water column. Importantly, this could also indicate that MC biodegradation was not occurring. If the latter is true, this highlights the crucial need for routine detection and implementation of appropriate water treatments as biodegradation of MCs may not be as universally important as previously thought.

## Data Availability Statement

The information to access the datasets generated and analyzed in this study can be found in section “Materials and Methods” or Supplementary Tables S3, S4.

## Author Contributions

LK, GLB, and SW designed the study. MS extracted RNA from *Taihu* in 2013. GSB and RM provided the data from the Lake Erie diel study. LK analyzed the metatranscriptomes. All authors drafted the manuscript.

## Conflict of Interest

The authors declare that the research was conducted in the absence of any commercial or financial relationships that could be construed as a potential conflict of interest.

## Footnotes

1 matsen.fhcrc.org/pplacer/
