# Supplementary material for: Insight Into the Molecular Mechanisms for Microcystin Biodegradation in Lake Erie and Lake Taihu
Source: Front Microbiol. 2019 Dec 10;10:2741. doi: 10.3389/fmicb.2019.02741 (PMC6914704; doi:10.3389/fmicb.2019.02741)
Supplement: Supplementary file 1 [file Presentation_1.pdf]

SUPPLEMENTAL INFORMATION

TITLE: INSIGHT INTO THE MOLECULAR MECHANISMS FOR MICROCYSTIN BIODEGRADATION IN LAKE ERIE AND LAKE TAIHU

Lauren E. Krausfeldt[1], Morgan M. Steffen[2], Robert M. McKay[3], George S. Bullerjahn[4], Gregory L. Boyer[5], Steven W. Wilhelm[1]*

[1]University of Tennessee, Department of Microbiology, Knoxville, TN, USA

[2]James Madison University, Department of Biology, Harrisonburg, VA, USA

[3] Great Lakes Institute for Environmental Research, University of Windsor, Windsor, ON, Canada

[4]Bowling Green State University, Department of Biological Sciences, Bowling Green, OH, USA

[5]State University of New York, College of Environmental Science and Forestry, Department of Chemistry, Syracuse, NY, USA

*** Correspondence:**
Corresponding Author
wilhelm@utk.edu

Supplemental methods

Phylogenetic analysis of MlrA sequences

Protein sequences arising from the *mlrA* gene were downloaded from NCBI's GenBank and accession numbers are displayed in Figure 1. These sequences were aligned after eight iterations using the MUSCLE algorithm and a maximum likelihood tree was created in Mega v7.0 (Tamura *et al.*, 2007).

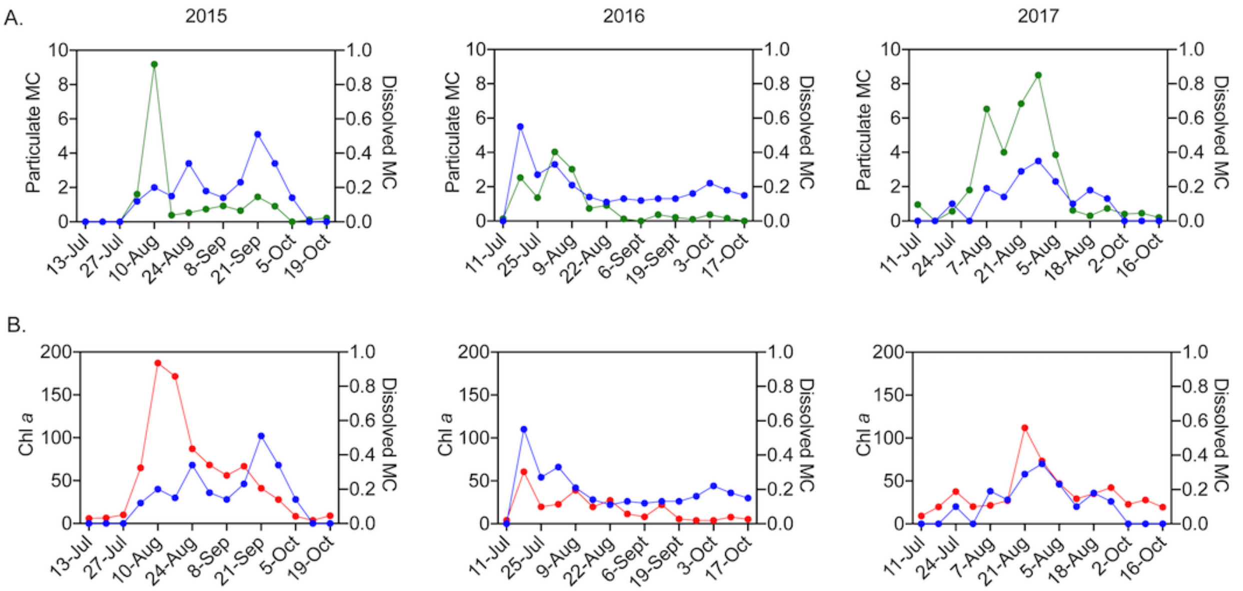

Figure S1. Dissolved and particulate MC dynamics during *Microcystis* blooms in Lake Erie. Data for dissolved and particulate MC concentrations and chl *a* were accessed through the NOAA Great Lakes Environmental Research Laboratory (https://www.glerl.noaa.gov/res/HABs_and_Hypoxia/WLEMicrocystin.html, accessed May 24, 2019).

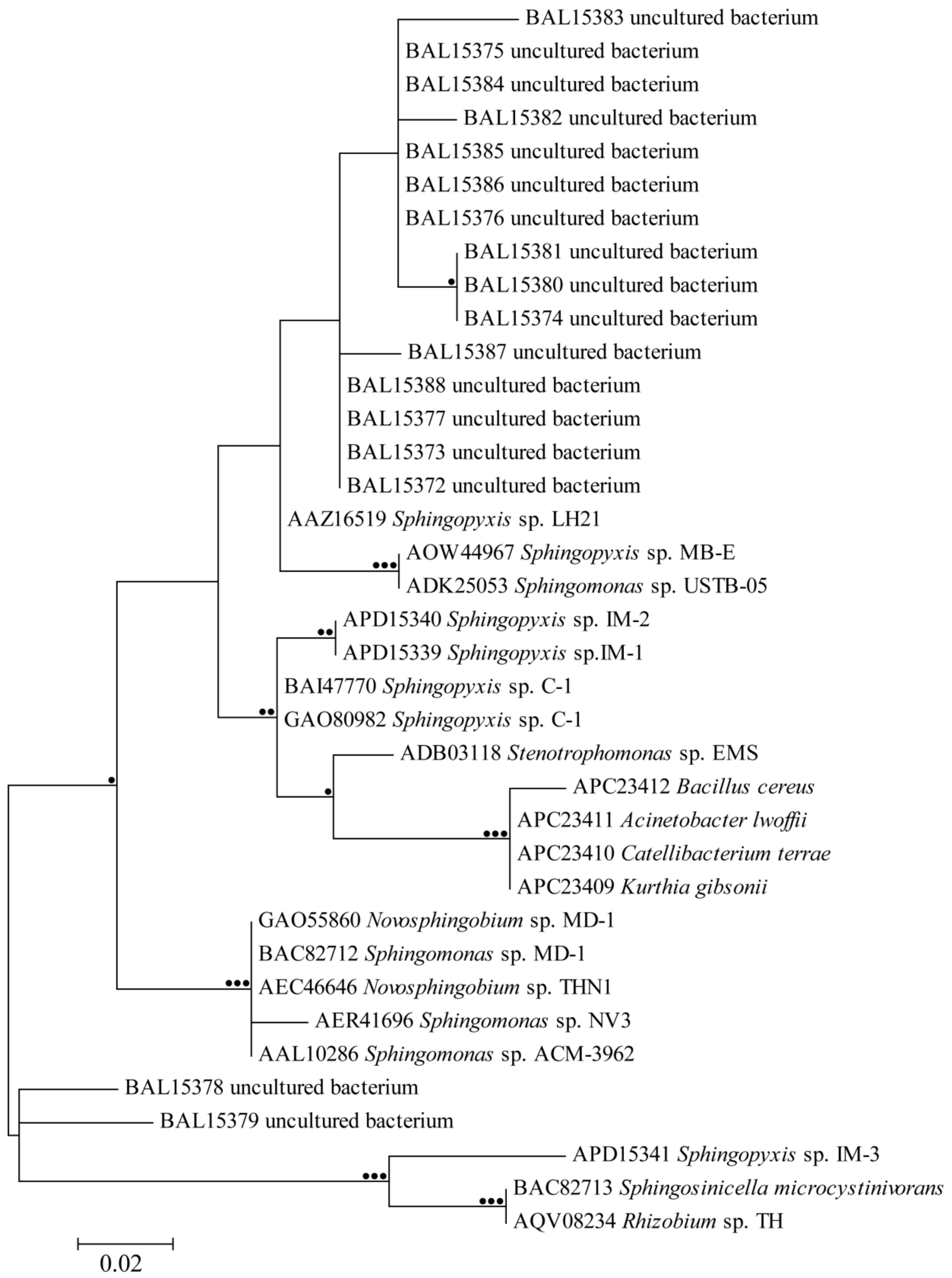

Figure S2. Maximum likelihood tree describing the phylogenetic distribution of MlrA protein sequences. Tree was bootstrapped 1000 times. Bootstrap values are represented by circles at nodes. ●●● >90, ●● >70, ● >50

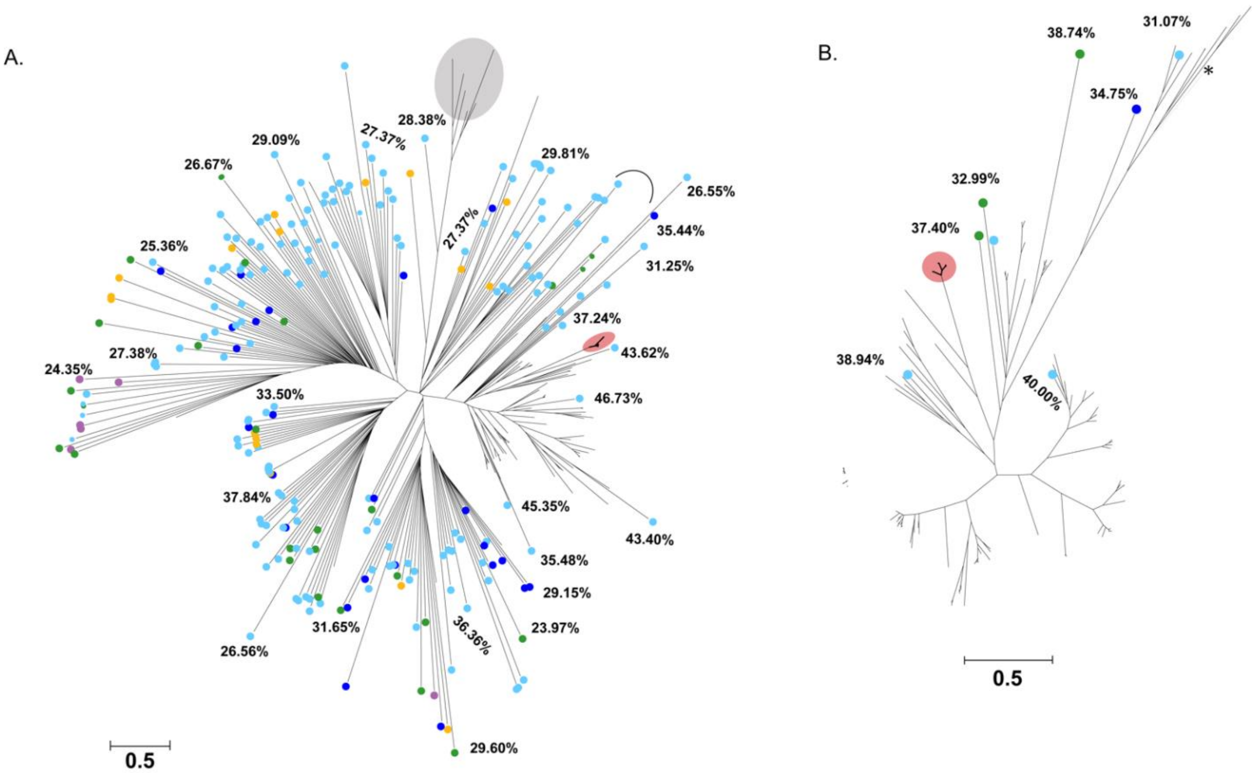

Figure S3. Maximum likelihood phylogenetic tree with contigs similar (defined as having an E value of <0.00001 using BLASTx) to MlrB (A) and MlrC (B). Colored closed circles represent contigs from each study aligned using pplacer: orange = Lake Erie 2013; light blue = Lake Erie Diel 2014; dark blue = Lake Erie 2014; purple = Lake Taihu 2014; green = Lake Taihu 2013. In A and B, the clades highlighted in red are MlrB and MlrC, respectively. The clade highlighted in gray in A represented beta-lactamases with confirmed function. The percentages indicate the greatest percent identities of select sequences from original BLASTx used for detecting MlrB and MlrC sequences.

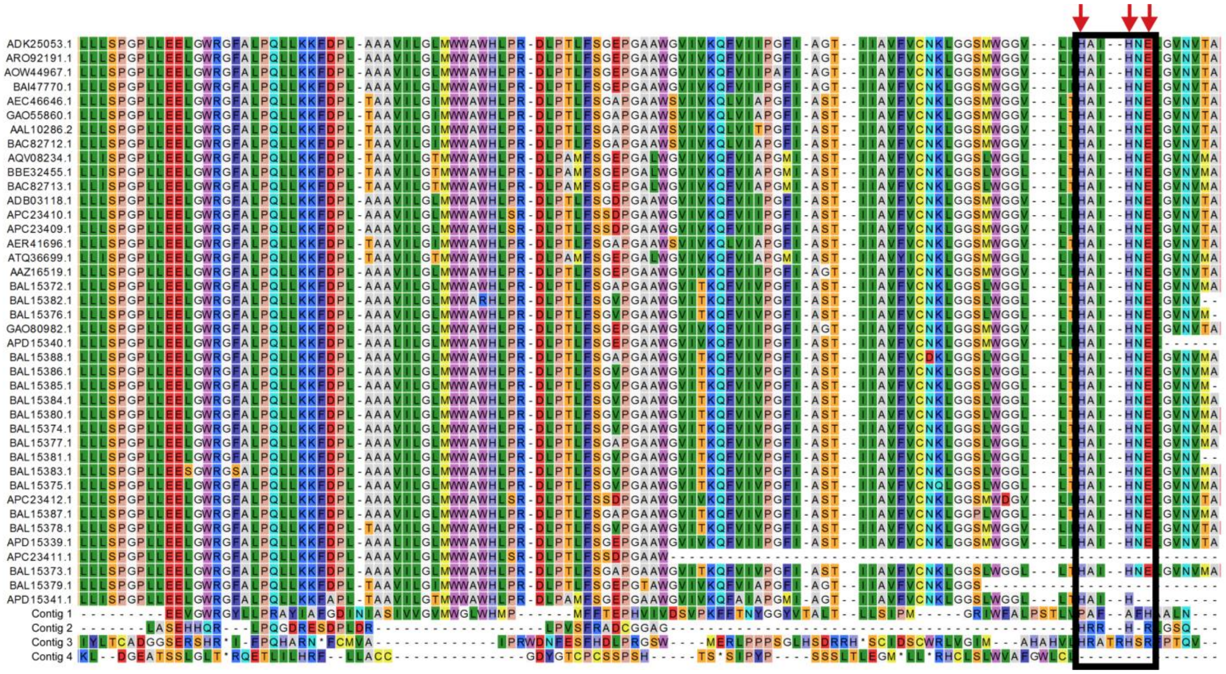

Figure S4. Alignment of MlrA reference sequences (indicated by accession number) used in this study and MlrA candidates identified. The black box highlights the active site proposed and the red arrows are necessary residues for functionality (Dziga et al. 2012). The alignment was performed in CLC Genomics Workbench using default parameters.

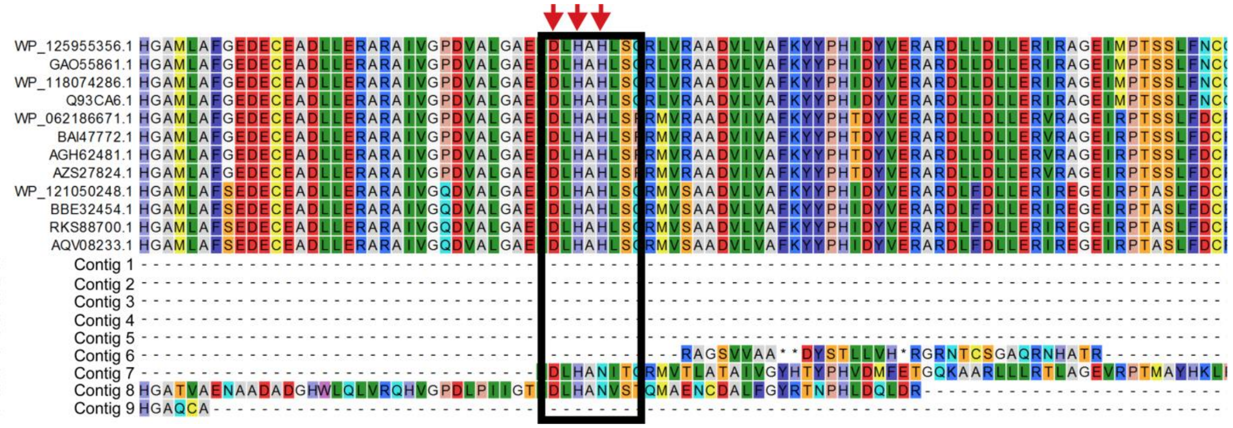

Figure S5. Alignment of MlrC reference sequences (indicated by accession number) used in this study and MlrC candidates identified. The black box highlights the active site proposed and the red arrows are necessary residues for functionality (Dziga et al., 2016). The alignment was performed in CLC Genomics Workbench using default parameters.

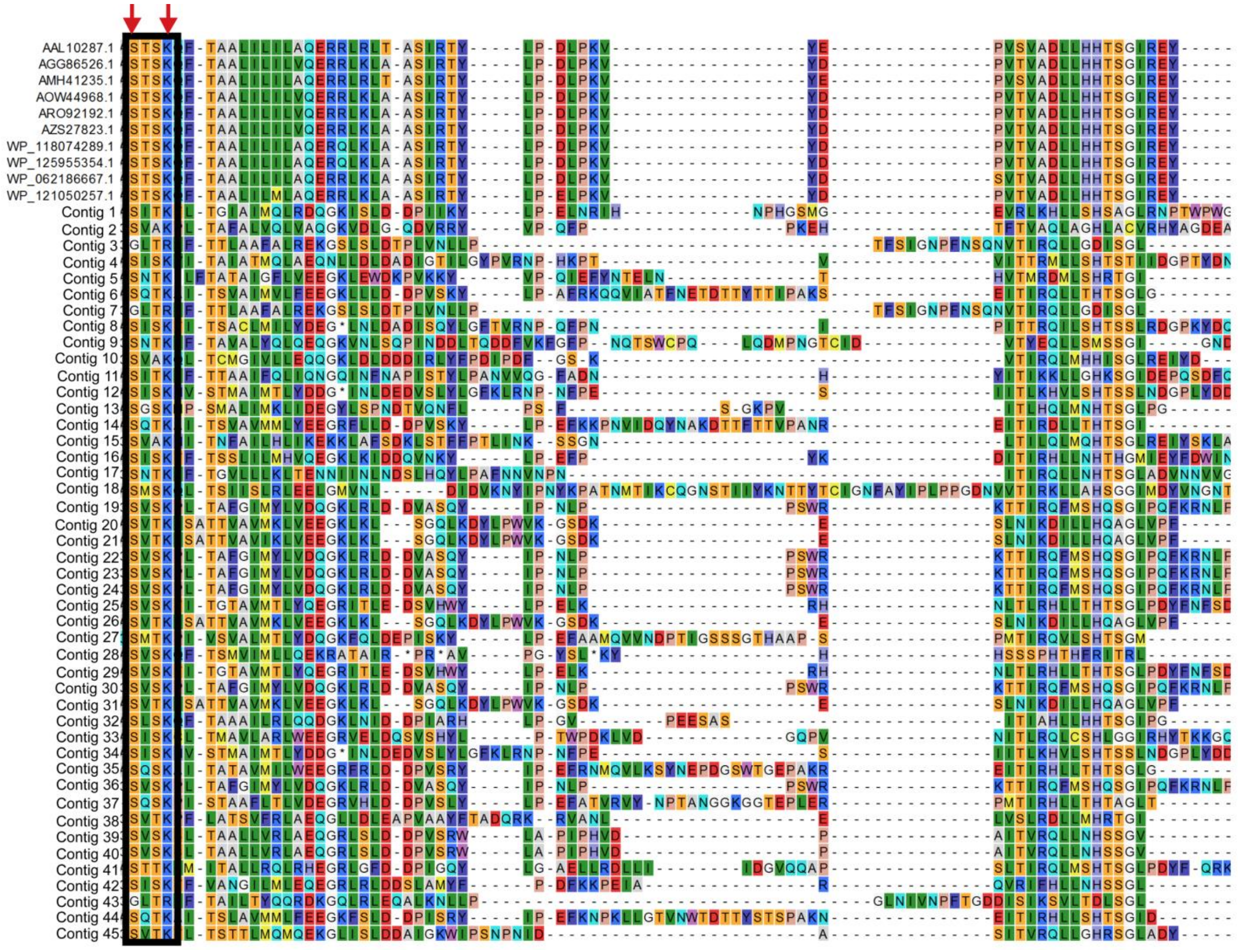

Figure S6. Alignment of MlrB reference sequences (indicated by accession number) used in this study and a subset of the MlrB candidates identified. The black box highlights the active site proposed and the red arrows are necessary residues for functionality (Dziga et al., 2016). The alignment was performed in CLC Genomics Workbench using default parameters.

Table S1. Microcystin degrading bacteria, their origin and the MC congeners that were degraded are listed below. The detection method for microcystin and whether or not *mlrA* or degradation productions were detected is also indicated.

Isolate	Site	Congener	*mlrA*	Reference
Sphingomonas sp. ACM3962	Murrumbidgee River, New South Wales	LR	yes[a]	(Jones *et al.*, 1994, Bourne *et al.*, 1996, Bourne *et al.*, 2001),
Pseudomonas aeruginosa	Ohi water purification facility, Japan	LR	-	(Takenaka & Watanabe, 1997)
Sphingosinicella microcystinivorans isolate Y2 (AB084247)	Lake Suwa, Japan	LR, 6(Z)-Adda LR, RR, YR	yes[b]	(Park *et al.*, 2001, Maruyama *et al.*, 2003, Saito *et al.*, 2003)
Sphingomonas sp. isolate MD-1 (AB110635)	Lake Kasumigaura, Japan	LR, RR, YR	yes[b]	(Saito *et al.*, 2003)
Sphingomonas sp. isolate 7CY (AB076083)	Lake Suwa, Japan	LR, LY, LW, LF. RR	-	(Ishii *et al.*, 2004)
Poterioochromonas (AY699607)	Culture of *Microcystis*	LR, RR	-	(Ou *et al.*, 2005)
Paucibacter toxivorans sp.	Lake Tuusulanjarvi, Finland	-	-	(Rapala *et al.*, 2005)
Cultivated microbes from GAC filter	-	LR, RR	-	(Lee *et al.*, 2006)
Sphingomonas sp. isolate B9 (AB159609)	Lake Sagami and Tsukui, Japan	LR, RR	-	(Tsuji *et al.*, 2006)
Sphingomonas sp. isolate CBA4 (AY920497)	San Roque reservoir, Argentina	RR	-	(Valeria *et al.*, 2006)
Sphingopyxis sp. isolate LH21 (DQ112242)	sand filter	LR, LA	yes[b]	(Ho *et al.*, 2007)
Bifidobacteria and lactobacilli strains	Culture collection and dadih	LR	no[c]	(Nybom *et al.*, 2007, Nybom *et al.*, 2008, Nybom *et al.*, 2012)
Bulkholderia sp. (DQ459360)	Patos Lagoon, Brazil	LR	-	(Lemes *et al.*, 2008)
Poterioochromonas isolate ZX1 (EF165114)	-	LR	-	(Zhang *et al.*, 2008)
Sphingopyxis sp. isolate C-1 (AB161684)	Hongfeng Lake, China	LR	yes[b]	(Okano *et al.*, 2009)
Methylobacillus sp. isolate J10 (FJ418599)	Lake Taihu sludge, China	LR, RR	-	(Hu *et al.*, 2009)
Arthrobacter sp., *Brevibacterium* sp., *Rhodococcus* sp.	Loch Rescobie, United Kingdom	LR, RR, LF, LW, LY	no	(Manage *et al.*, 2009, Lawton *et al.*, 2011)
Sphinogpyxis sp. isolate USTB-05 (EF607053)	Lake Dianchi, China	RR	yes[d]	(Junfeng *et al.*, 2010, Zhang *et al.*, 2010) (Yan *et al.*, 2012, Yan *et al.*, 2012)
Morganella morganii (LAAFP-C25216), *Pseudomonas* sp. (*Sphingomonas* C25358 and Lake Mead C25459)	Lake Mead and Los Angeles Aqueduct Filtration Plant	LR	-	(Eleuterio & Batista, 2010)
Stenotrophomonas sp. isolate EMS (FJ712028)	Lake Taihu sludge, China	LR, RR	yes[b]	(Chen *et al.*, 2010)
Bacillus sp. isolate AMRI-03 (GU294753)	Tendaha Lake, Saudia Arabia	RR	yes[b]	(Alamri, 2010)
Ralstonia solanacearum	Lake Dianchi, China	LR	-	(Zhang *et al.*, 2011)
Microbacterium sp. isolate DC8 and *Rhizobium gallicum* isolate DC7 (AY972457)	Lake Okeechobee	LR	-	(Ramani *et al.*, 2012)
Novosphingobium sp. isolate THN1 (HQ664117)	Lake Taihu, China	LR	yes[e]	(Jiang *et al.*, 2011)
Trichaptum abietinum isolate1302BG	bamboo forest, China	LR	-	(Jia *et al.*, 2012)
Bacillus flexus isolate SSZ01 (GU112451)	Tendaha Lake, Saudi Arabia	RR	yes[b]	(Alamri, 2012)
Bacillus sp. isolate EMB (FJ526332)	Soil from heap of algae, China	LR, RR	yes[b]	(Hu *et al.*, 2012)
Pseudomonas aeruginosa PA14 and *Pseudomonas putida* KCCM10464	Culture Collection	-	-	(Kang *et al.*, 2012)
Sphingopyxis sp. isolate TT25 (JQ398614)	Myponga Reservoir	LR, RR, YR, LA	yes[f]	(Ho *et al.*, 2012)
Novosphingobium sp. isolate KKU03	Nong Kin Moo, Khon Kaen, Thailand	LR, [Dha[7]] LR	-	(Somdee *et al.*, 2013)
Isolate KKU-12	Kaen Nakhon Lake, Thailand	-	-	(Phujomjai & Somdee, 2013)
Sphingmonas sp. isolate NV3	Lake Rotoiti, New Zealand	[Dha[7]] LR	yes[b]	(Somdee *et al.*, 2013)
Ochrobactrum sp. isolate FDT5	activated sludge	LR	-	(Jing *et al.*, 2014)
Portulaca oleracea cv.	-	LR	-	(Isobe *et al.*, 2014)
Bordetella petrii (KC7348821.1)	Lake Taihu sludge	LR, RR	yes[b]	(Yang *et al.*, 2014)

[a] : Fosmid library; [b]: Amplification, Saito et al. 2003 [c]: Amplification, Wang; [d]: Genomic searches [e]: Amplification, Jiang 2014; [f]: Amplification, Hoefel 2009; [g]: Amplification, Ho 2007; [h]: Qin 2019

Table S1 continued.

Isolate	Site	Congeners	*mlrA*	Reference
Stenotrophomonas acidaminiphila isolate MC LTH2 (KF305533)	Lake Taihu sludge	LR, RR	no[b]	(Yang *et al.*, 2014)
Trichoderma citronoviride isolate kkuf-0955	Saudi eutrophic lake	-	-	(Mohamed *et al.*, 2014)
Pseudomonas sp. isolates WC-5, WC-4	Lake Taihu sediment, China	LR, RR	-	(Li & Pan, 2014)
Bacillus nanhaiencis isolate JZ-2013 (KF841622)	Lake Chaohu China	LR	-	(Zhang *et al.*, 2015)
Arthobacter sp. isolate T11	Lake Taoranting sediment, China	LR, RR	-	(Qu *et al.*, 2015)
Aeromonas veronii isolate w-s-03 (JF490063.1)	Sulejow Reservoir, Poland	LR	no[b]	(Mankiewicz-Boczek *et al.*, 2015)
Psuedomonas aeruginosa isolate DMXS	Palos Lagoon sediment, Brazil	LR	-	(Lemes *et al.*, 2015)
Steroidobacter flavus	forest soil, China	LR	-	(Gong *et al.*, 2016)
Rhizobium sp. isolate TH (KM365438)	Lake Taihu sediment	LR	yes[b]	(Zhu *et al.*, 2016)
Novosphingobium sp. Isolate KKU25s	Bueng nong Khot reservoir, Thailand	[Dha[7]] LR	yes[b]	(Phujomjai *et al.*, 2016)
anaerobic amino acid degrading bacteria Ala-1 (DSM 12261)	DSM, Germany	LR	no[b]	(Bao & Wu, 2016)
Sphingopyxis sp isolates IM-1 (KX085478), IM-2 (KX085479), IM-3 (KX085480)	Pond water in Alberche's River, Spain	LR, YR	yes[g]	(Lezcano *et al.*, 2016)
Paucibacter toxinivorans isolate IM-4 (KX085481)	forest soil, China	LR	no[g]	(Gong *et al.*, 2016)
Bacillus sp.	Hulupi Lake, Taiwan	LR	no	(Kansole & Lin, 2016)
Sphingopyxis sp. Isolate a7 (NR_113720)	Lake Taihu, China, sludge	MC-LR	yes[b]	(Zhang et al., 2017)
Citrobacter sp. Isolate S06 (MC586881), *Serratia* sp. Isolate S05 (MG586880)	Water from Lake Mikolajski, Poland	MC-LR, MC-RR	n/a	(Bukowska *et al*., 2018)
Citrobacter sp. Isolate W03 (MG586884)	Sediment from Lake Mikolajski, Poland	LR, YR		(Lezcano *et al.*, 2016)
Rahnella aquaticus isolate 13UL (KJ954305)	Freshwater reservoir Ulhitiya, Sri Lanka	MC-LR	yes[b]	(Idroos & Manage, 2018)
Bacillus cereus isolate 12GK (KJ954304)	Freshwater reservoir Girandurukotte	LR	yes[b]	(Lee et al., 2018)
Pseudomonas sp. isolate R12, isolate S42, *and Pantoea* sp. isolate *S65*	Flower garden soil, Korea	n/a	n/a[b*]	
Pseudomonas putida isolates LEw-1033 (KX185385), LEw-2166 (KX185392), *Stenotrophomonas maltophila* isolate LEw-1278 (KX185597), *Bacillus thuringiensis* isolate LEw-2010 (KX185387), *Brevibacillus brevis* isolate LEw-1238 (KX185386), Acidovorax facilis isolate LEw-2 (KX753361)	Surface water Lake Erie, USA	MC-LR	no[b]	*(*Krishnan *et al.*, 2018*)*
Sphingopyxis granuli Kw07 (isolate m6, MF535105))	Salvage yard water in Fuda bay, Lake Taihu, China	MC-LR	yes[b]	(Ding *et al*., 2018)
Isolate X20 (KM365437)	Sediment from Lake Dianchi, China	MC-LR	yes[b]	(Qin *et al*., 2019)
Novosphingobium resinovorum isolate GR1 GB, and *Variovorax* sp. isolate SL OY	Water from Lake Erie, USA	MC-LR	no[b]	(Thees *et al*., 2019)

[a] : Fosmid library; [b]: Amplification, Saito et al. 2003 [c]: Amplification, Wang; [d]: Genomic searches [e]: Amplification, Jiang 2014;
[f]: Amplification, Hoefel 2009; [g]: Amplification, Ho 2007; [h]: Qin 2019
*: based only on PCR band length

Table S2. Environmental data collected for *Taihu* 2013 metatranscriptomes collected on 8/8/2013. Stn = Taihu station number; EC = electric conductivity; TDS = total dissolved solids; NTU = turbidity; DO = dissolved oxygen

Sample ID	Latitude/ Longitude	Stn	Sampling Time	Water temp (°C)	EC (µs/cm)	TDS (g/L)	Sal (‰)	pH	NTU	DO (%)	DO (mg/L)
mgm4768724	31.40/120.03	3	13:06	33.5	693	0.387	0.28	8.8	39.7	124	8.83
mgm47687228	31.48/120.19	6	13:28	33.74	692	0.386	0.28	8.8	74.4	118.4	8.37
mgm4768720	31.45/120.12	Ch	13:46	33.74	636	0.354	0.26	8.7	73.8	132.7	9.41
mgm4768721	31.35/120.33	17A	14:25	34.21	570	0.315	0.23	9.4	159.2	213.2	14.96
mgm4768726	31.42/120.22	31	15:13	35.07	714	0.39	0.28	9	23.2	144.1	9.74
mgm47687230	31.39/120.30	13	15:36	35.01	714	0.391	0.29	8.9	51.3	119.3	8.45

Table S3. Information for published metatranscriptomes from *Taihu* screened for microcystin degradation genes. Values represent the number of transcripts that mapped to putative MC degradation genes or the *mcyD* from *M. aeruginosa* normalized by contig length and per 1,000,000 transcripts for each library. GST=glutathione disulfide; AP = alkaline protease; Chla = chlorophyll a; MC = total particulate microcystin

MG-RAST Sample ID	Date	Latitude/ Longitude	Station	Total trimmed reads	*mcyD*	*mlrA*	GST	AP	Chla (ug/L)	MC (ug/L)
mgm4690174	8/8/13	31.40/120.03	Stn 17	48863932	0.002273	0	0	0	n/a	n/a
mgm4690179	8/8/13	31.48/120.19	Stn 3	55389670	0.004318	0	1.55733	0.056689	n/a	n/a
mgm4690175	8/8/13	31.45/120.12	Channel	46161784	0.011474	0	0	0	n/a	n/a
mgm4690178	8/8/13	31.35/120.33	Stn 31	70625448	0.00254	0	1.804874	0	n/a	n/a
mgm4690177	8/8/13	31.42/120.22	Dock	70749456	0.001811	0.031096	0.359013	0.067562	n/a	n/a
mgm469176	8/8/13	31.39/120.30	Stn 13	52794692	0.001942	0	0.840804	0	n/a	n/a
mgm4808410	6/7/14	31.51/120.19	Stn 1	22882524	0.020533	0	0.419534	0	40.176	0.28
mgm4808428	6/7/14	31.42/120.22	Dock	25222008	0.514143	0	0.115375	0	959.76	69.39
mgm4808444	6/7/14	31.39/120.23	Stn 328	24028518	0.155718	0	0.144828	3.684372	370.2888	7.18
mgm4808429	6/7/14	31.44/120.19	Stn 4	24699454	0.030782	0	0.245754	0	54.0144	1.18
mgm4808417	6/7/14	31.45/120.12	Channel	28729970	0.034789	0	0.346676	0	368.28	2.64
mgm4808447	6/7/14	31.34/120.18	Stn 7	13778860	0.00496	0	0.112491	0.100807	27.342	0
mgm4808438	6/7/14	31.31/119.95	Stn 19	18348450	0.037246	0	0.068126	0	48.2112	3.63
mgm4808440	7/3/14	31.51/120.19	Stn 1	14223218	0.152555	0	0.165926	0	18.7488	0.24
mgm4808426	7/3/14	31.42/120.22	Dock	22362264	0.100087	0	0.146228	0	119.3283	0.24
mgm4808446	7/3/14	31.39/120.23	Stn 328	23410036	0.537882	0	0.237078	0.355958	46.872	5.55
mgm4808432	7/3/14	31.44/120.19	Stn 4	27244752	0.142979	0	0.441553	0	319.92	9.76
mgm4808433	7/3/14	31.45/120.12	Channel	23473470	0.098624	0	0.039619	0	38.13	2.96
mgm4808442	7/3/14	31.45/120.03	Stn 16	26556856	0.186892	0	0.086381	0	40.6224	0.39
mgm4808422	7/3/14	31.40/120.03	Stn 17	29089776	0.96733	0	0.115161	0	42.408	5.78
mgm4808414	7/3/14	31.31/119.95	Stn 10	32370808	0.001319	0	0.898031	0	15.252	0.21
mgm4808425	8/14/14	31.51/120.19	Stn 1	25763998	0.151197	0	0.799177	0	74.5767	2.71
mgm4808423	8/14/14	31.42/120.22	Stn 328	24398486	0.299011	0	0.266164	0	71.61372	2.53
mgm4808445	8/14/14	31.44/120.19	Stn 4	22900642	0.143617	0	0.220518	0.05895	89.2242	2.76
mgm4808441	8/14/14	31.45/120.12	Channel	20641288	0.335228	0	0	0	3257.604	111.57
mgm4808430	8/14/14	31.40/120.03	Stn 17	22209250	0.258865	0	0.260252	0	100.719	6.58
mgm4808439	8/14/14	31.31/119.95	Stn 10	24413932	0.005948	0	2.175397	0.023347	21.11472	0.28
mgm4808421	9/9/14	31.51/120.19	Stn 1	26102478	0.157418	0	0.327938	0.078537	57.1392	0.45
mgm4808419	9/9/14	31.39/120.23	Stn 328	23315430	0.139962	0	0.218739	8.146965	249.5934	2.8
mgm4808409	9/9/14	31.44/120.19	Stn 4	25593264	0.107812	0	0.520449	0	50.8896	0

mgm4808436	9/9/14	31.45/120.12	Channel	24661602	0.404242	0	0.032845	0	16182	592.69
mgm4808424	9/9/14	31.45/120.03	Stn 16	25189504	0.59145	0	0.214772	0	3414.96	30.89
mgm4808413	9/9/14	31.40/120.19	Stn 7	24005774	0.389308	0	0.033742	14.19867	1204.722	75.04
mgm4808418	9/9/14	31.31/119.95	Stn 17	26688906	1.239036	0	0.482223	0	307.1232	2.54
mgm4808412	10/8/14	31.51/120.19	Stn 1	34238048	0.297163	0	0.176412	0.020737	32.587	0.19
mgm4808411	10/8/14	31.42/120.22	Dock	25094068	0.933784	0	0.712917	0	1037.88	0.62
mgm4808427	10/8/14	31.39/120.23	Stn 328	23832264	0.259875	0	0.26225	0.609258	1622.106	348.51
mgm4808437	10/8/14	31.44/120.19	Stn 4	31306012	0.366744	0	0.176005	0	29.686	0
mgm4808415	10/8/14	31.45/120.03	Stn 16	26862182	0.306886	0	0.078177	0	37.4976	0.93
mgm4808434	10/8/14	31.40/120.19	Stn 7	42067072	0.337302	0	0.404592	0	1507.577	298.08
mgm4808443	10/8/14	31.31/119.95	Stn 10	15867008	0.324649	0	0.066805	0	97.5384	0.24

Table S4. Information for published metatranscriptomes from Lake Erie screened for microcystin degradation genes. Values represent the number of transcripts that mapped to putative MC degradation genes or the *mcyD* from *M. aeruginosa* normalized by contig length and per 1,000,000 transcripts for each library. GST=glutathione disulfide; AP = alkaline protease; Chla = chlorophyll a; MC = total particulate microcystin

**MG-RAST Sample ID**	**Date**	**Latitude/ Longitude**	**Station**	**Total trimmed reads**	***mcyD***	***mlrA***	**GST**	**AP**	**Chla (ug/L)**	**MC (ug/L)**
mgm4559605	8/15/12	41.76/-83.30	Stn 973	47191952	0.0009051	0	0	0	25.4	0.37
mgm4559606	8/15/12	41.76/-83.30	Stn 973	47731372	0.0028636	0	0	0	25.4	0.37
mgm4559607	8/15/12	41.76/-83.30	Stn 973	32936224	0.0028531	0	0	0	25.4	0.37
mgm4559602	8/15/12	41.78/-83.33	Stn 882	43562732	0.0013727	0	0	0	16.1	0.11
mgm4559603	8/15/12	41.78/-83.33	Stn 882	48714106	0.0001754	0	0	0	16.1	0.11
mgm4559604	8/15/12	41.78/-83.33	Stn 882	41936582	0.0008148	0	0	0	16.1	0.11
mgm4559599	8/14/12	41.47/-82.72	Stn 1163	469011364	1.821E-05	0	0	0	15.6	0.61
mgm4559600	8/14/12	41.47/-82.72	Stn 1163	47554166	0	0	0	0	15.6	0.61
mgm4559601	8/14/12	41.47/-82.72	Stn 1163	46661034	0.0001831	0	0	0	15.6	0.61
152a23ca156d 676d3438313 13039342e33	7/24/13	41.78/-83.33	Stn 882	53933774	0.1960881	0	0.0574779	0.0189121	6.54	0.13
f7052ec2616d 676d3438313 13039322e33	7/24/13	41.78/-83.33	Stn 882	60539408	0.1745513	0	0.0771398	0	6.54	0.13
c2372475796d 676d3438313 13038352e33	7/24/13	41.78/-83.33	Stn 882	80313098	0.1719947	0	0.0647466	0	6.54	0.13
d1e3fa92a66d 676d3438313 13038372e33	7/24/13	41.76/-83.30	Stn 973	74370184	0.1945835	0	0.0294473	0	3.44	0.07
9d8616f47e6d 676d3438313 13039302e33	7/24/13	41.76/-83.30	Stn 973	103691402	0.1899801	0	0	0	3.44	0.07
mgm4813850	8/26/14	41.70/-83.25	n/a	122728593	0.1756156	0.0488884	0.672215	0.1679315	31.02	1.54
mgm4813853	8/27/14	41.70/-83.25	n/a	133304562	0.0349896	0.0159784	0.2369761	0	32.39	1.07
mgm4813847	8/27/14	41.70/-83.25	n/a	125015543	0.0787191	0	0.9790783	0.1648595	38.56	1.29
mgm4813849	8/27/14	41.69/-83.25	n/a	131515535	0.1751844	0.022811	0.7523066	0.0656957	42.68	2.68
mgm4813852	8/27/14	41.69/-83.25	n/a	120490748	0.1093257	0.0199185	0.6602997	0.0328656	44.1	1.50
mgm4813848	8/28/14	41.70/-83.27	n/a	130523790	0.060082	0	0.7497484	0.121587	54.15	4.92
mgm4813851	8/28/14	41.70/-83.29	n/a	149931718	0.5756935	0	0.1776809	0.0354161	54.34	3.62
mgm4690168	8/4/14	41.75/-83.32	WE12	11629072	0.1469185	0	0.3078492	0	54.46	2.91
mgm4690167	8/4/14	41.75/-83.32	WE12	21589946	0.2354273	0	0.6780934	0.1139419	54.46	2.91
mgm4690161	8/4/14	41.83/-83.18	W8	23284722	0.140147	0	1.3120191	0	46.53	1.1
mgm4690159	8/4/14	41.67/-83.23	EOI	23916440	0.2493161	0	0.6447448	0.1697577	69.44	1.24

mgm4690164	8/4/14	41.82/-83.18	W4	25537490	0.087977	0	1.2217332	1.0572691	18.11	0.52
mgm4690155	8/4/14	41.75/-83.32	W2	24352730	0.0564768	0	0.7009481	0.2632148	29.44	0.91
mgm4690684	8/4/14	41.73/-83.13	WE13	25112974	0.2537653	0.2373275	0.5901332	0.5367743	6.74	0.4
mgm4690169	8/4/14	41.75/-83.32	W2	39786886	0.0918957	0	0.5934116	0.0940008	29.44	0.91
mgm4690158	8/4/14	41.70/-83.37	W6	36355044	0.0782476	0	1.2303657	0	71.62	3.45
mgm4690166	8/4/14	41.72/-83.33	WOI	22147772	0.3833962	0	0.801435	1.583455	51.2	1.1
mgm4690157	8/4/14	41.72/-83.33	WOI	27507218	0.2357147	0	0	2.0969042	51.2	1.1
mgm4690156	8/4/14	41.82/-83.18	w4	16574284	0.0742197	0	0.7354767	0	18.11	0.52
mgm4690162	8/4/14	41.67/-83.23	EOI	29261666	0.1115208	0	1.1581706	0	69.44	1.24
mgm4690160	8/4/14	41.75/-83.32	w12	20773486	0.0464687	0	0.2036249	0	54.46	2.91
mgm4690683	8/4/14	41.73/-83.13	w13	29786532	0.1998961	0	0.6036285	0	6.74	0.4
mgm4690154	8/4/14	41.75/-83.32	w12	21334578	0.0092095	0	0	0	54.46	2.91

Table S5. Number of reads mapped to the *mlr*ABCD genes of other MC degrading species. n/a = no reads mapped

	Taihu 2014	Lake Erie 2014	Diel 2014	Taihu 2013	Lake Erie 2012	Lake Erie 2013
Novosphingobium sp. THN1	15	27	n/a	n/a	n/a	n/a
Rhizobium sp. TH	9	4	n/a	1	n/a	n/a
Sphingopyxis sp. C-1	9	3	6	n/a	n/a	n/a

References

Alamri S. A. 2010. Biodegradation of microcystin by a new *Bacillus* sp. isolated from a Saudi freshwater lake. *African Journal of Biotechnology*. 9, 6552-6559.

Alamri S. A. 2012. Biodegradation of microcystin-RR by *Bacillus flexus* isolated from a Saudi freshwater lake. *Saudi Journal of Biological Sciences*. 19, 435-440.

Bao Z. & Wu Y. 2016. Biodegradation of microcystin-LR by an amino acid-degrading anaerobic bacterium. *Desalination and Water Treatment*. 57, 870-880.

Bourne D. G., Riddles P., Jones G. J., Smith W. & Blakeley R. L. 2001. Characterisation of a gene cluster involved in bacterial degradation of the cyanobacterial toxin microcystin LR. *Environ Toxicol*. 16, 523-534.

Bourne D. G., Jones G. J., Blakeley R. L., Jones A., Negri A. P. & Riddles P. 1996. Enzymatic pathway for the bacterial degradation of the cyanobacterial cyclic peptide toxin microcystin LR. *Applied and Environmental microbiology*. 62, 4086-4094.

Chen J., Hu L. B., Zhou W., Yan S. H., Yang J. D., Xue Y. F. & Shi Z. Q. 2010. Degradation of microcystin-LR and RR by a *Stenotrophomonas* sp. strain EMS isolated from Lake Taihu, China. *International journal of molecular sciences*. 11, 896-911.

Eleuterio L. & Batista J. R. 2010. Biodegradation studies and sequencing of microcystin-LR degrading bacteria isolated from a drinking water biofilter and a fresh water lake. *Toxicon*. 55, 1434-1442.

Gong Z.-L., Zhang C.-F., Jin R. & Zhang Y.-Q. 2016. Steroidobacter flavus sp. nov., a microcystin-degrading Gammaproteobacterium isolated from soil. *Antonie van Leeuwenhoek*. 109, 1073-1079.

Ho L., Hoefel D., Saint C. P. & Newcombe G. 2007. Isolation and identification of a novel microcystin-degrading bacterium from a biological sand filter. *Water Res*. 41, 4685-4695.

Ho L., Tang T., Monis P. T. & Hoefel D. 2012. Biodegradation of multiple cyanobacterial metabolites in drinking water supplies. *Chemosphere*. 87, 1149-1154.

Hu L., Zhang F., Liu C. & Wang M. 2012. Biodegradation of Microcystins by *Bacillus* sp. strain EMB. *Energy Procedia*. 16, 2054-2059.

Hu L. B., Yang J. D., Zhou W., Yin Y. F., Chen J. & Shi Z. Q. 2009. Isolation of a *Methylobacillus* sp. that degrades microcystin toxins associated with cyanobacteria. *N Biotechnol*. 26, 205-211.

Ishii H., Nishijima M. & Abe T. 2004. Characterization of degradation process of cyanobacterial hepatotoxins by a Gram-negative aerobic bacterium. *Water Res*. 38, 2667-2676.

Isobe T., Okuhata H., Miyasaka H., Jeon B.-S. & Park H.-D. 2014. Detoxification of microcystin-LR in water by *Portulaca oleracea* cv. *Journal of bioscience and bioengineering*. 117, 330-332.

Jia Y., Du J., Song F., Zhao G. & Tian X. 2012. A fungus capable of degrading microcystin-LR in the algal culture of *Microcystis aeruginosa* PCC7806. *Applied biochemistry and biotechnology*. 166, 987-996.

Jiang Y., Shao J., Wu X., Xu Y. & Li R. 2011. Active and silent members in the *mlr* gene cluster of a microcystin-degrading bacterium isolated from Lake Taihu, China. *FEMS microbiology letters*. 322, 108-114.

Jing W., Sui G. & Liu S. 2014. Characteristics of a microcystin-LR biodegrading bacterial isolate: Ochrobactrum sp. FDT5. *Bulletin of environmental contamination and toxicology*. 92, 119-122.

Jones G. J., Bourne D. G., Blakeley R. L. & Doelle H. 1994. Degradation of the cyanobacterial hepatotoxin microcystin by aquatic bacteria. *Natural toxins*. 2, 228-235.

Junfeng W., Pengfei W., Jian C. & Hai Y. 2010. Biodegradation of microcystin-RR by a new isolated *Sphingopyxis* sp. USTB-05. *Chinese Journal of Chemical Engineering*. 18, 108-112.

Kang Y.-H., Park C.-S. & Han M.-S. 2012. *Pseudomonas aeruginosa* UCBPP-PA14 a useful bacterium capable of lysing *Microcystis aeruginosa* cells and degrading microcystins. *Journal of Applied Phycology*. 24, 1517-1525.

Kansole M. M. & Lin T.-F. 2016. Microcystin-LR Biodegradation by *Bacillus* sp.: Reaction Rates and Possible Genes Involved in the Degradation. *Water*. 8, 508.

Lawton L., Welgamage A., Manage P. & Edwards C. 2011. Novel bacterial strains for the removal of microcystins from drinking water. *Water Science and Technology*. 63, 1137-1142.

Lee Y.-J., Jung J.-M., Jang M.-H., Ha K. & Joo G.-J. 2006. Degradation of microcystins by adsorbed bacteria on a granular active carbon(GAC) filter during the water treatment process. *Journal of Environmental Biology*. 37, 317-322.

Lemes G. A., Kist L. W., Bogo M. R. & Yunes J. S. 2015. Biodegradation of [D-Leu 1] microcystin-LR by a bacterium isolated from sediment of Patos Lagoon estuary, Brazil. *Journal of Venomous Animals and Toxins including Tropical Diseases*. 21, 4.

Lemes G. A., Kersanach R., Pinto Lda S., Dellagostin O. A., Yunes J. S. & Matthiensen A. 2008. Biodegradation of microcystins by aquatic *Burkholderia* sp. from a South Brazilian coastal lagoon. *Ecotoxicol Environ Saf*. 69, 358-365.

Lezcano M. Á., Morón-López J., Agha R., López-Heras I., Nozal L., Quesada A. & El-Shehawy R. 2016. Presence or absence of *mlr* genes and nutrient concentrations co-determine the microcystin biodegradation efficiency of a natural bacterial community. *Toxins*. 8, 318.

Li H. & Pan G. 2014. Enhanced and continued degradation of microcystins using microorganisms obtained through natural media. *Journal of microbiological methods*. 96, 73-80.

Manage P. M., Edwards C., Singh B. K. & Lawton L. A. 2009. Isolation and identification of novel microcystin-degrading bacteria. *Appl Environ Microbiol*. 75, 6924-6928.

Mankiewicz-Boczek J., Gagala I., Jurczak T., Jaskulska A., Pawelczyk J. & Dziadek J. 2015. Bacteria homologus to *Aeromonas* capable of microcystin degradation. *Open Life Sci*. 10, 119-129.

Maruyama T., Kato K., Yokoyama A., Tanaka T., Hiraishi A. & Park H.-D. 2003. Dynamics of microcystin-degrading bacteria in mucilage of Microcystis. *Microbial Ecology*. 46, 279-288.

Mohamed Z. A., Hashem M. & Alamri S. A. 2014. Growth inhibition of the cyanobacterium *Microcystis aeruginosa* and degradation of its microcystin toxins by the fungus *Trichoderma citrinoviride*. *Toxicon*. 86, 51-58.

Nybom S., Dziga D., Heikkilä J., Kull T., Salminen S. & Meriluoto J. 2012. Characterization of microcystin-LR removal process in the presence of probiotic bacteria. *Toxicon*. 59, 171-181.

Nybom S. M., Salminen S. J. & Meriluoto J. A. 2007. Removal of microcystin-LR by strains of metabolically active probiotic bacteria. *FEMS microbiology letters*. 270, 27-33.

Nybom S. M., Collado M. C., Surono I. S., Salminen S. J. & Meriluoto J. A. 2008. Effect of glucose in removal of microcystin-LR by viable commercial probiotic strains and strains isolated from dadih fermented milk. *Journal of agricultural and food chemistry*. 56, 3714-3720.

Okano K., Shimizu K., Kawauchi Y., Maseda H., Utsumi M., Zhang Z., Neilan B. A. & Sugiura N. 2009. Characteristics of a Microcystin-Degrading Bacterium under Alkaline Environmental Conditions. *J Toxicol*. 2009, 954291.

Ou D., Song L., Gan N. & Chen W. 2005. Effects of microcystins on and toxin degradation by *Poterioochromonas* sp. *Environmental toxicology*. 20, 373-380.

Park H. D., Sasaki Y., Maruyama T., Yanagisawa E., Hiraishi A. & Kato K. 2001. Degradation of the cyanobacterial hepatotoxin microcystin by a new bacterium isolated from a hypertrophic lake. *Environmental toxicology*. 16, 337-343.

Phujomjai Y. & Somdee T. 2013. Isolation and characterization of microcystin-degrading bacterium. *Journal of Life Sciences and Technologies Vol*. 1,

Phujomjai Y., Somdee A. & Somdee T. 2016. Biodegradation of microcystin [Dha7] MC-LR by a novel microcystin-degrading bacterium in an internal airlift loop bioreactor. *Water Science and Technology*. 73, 267-274.

Qu J., Zhang Q., Zhang M., Xin Z., Li H. & Zhang N. 2015. Isolation of a *Arthrobacter* sp. T11 that Degrades Cyanobacterial Hepatotoxin Microcystin from the Sediment of a Shallow Hypertrophic Lake.

Ramani A., Rein K., Shetty K. & Jayachandran K. 2012. Microbial degradation of microcystin in Florida's freshwaters. *Biodegradation*. 23, 35-45.

Rapala J., Berg K. A., Lyra C., Niemi R. M., Manz W., Suomalainen S., Paulin L. & Lahti K. 2005. Paucibacter toxinivorans gen. nov., sp. nov., a bacterium that degrades cyclic cyanobacterial hepatotoxins microcystins and nodularin. *Int J Syst Evol Microbiol*. 55, 1563-1568.

Saito T., Okano K., Park H.-D., Itayama T., Inamori Y., Neilan B. A., Burns B. P. & Sugiura N. 2003. Detection and sequencing of the microcystin LR-degrading gene, *mlrA*, from new bacteria isolated from Japanese lakes. *FEMS Microbiology Letters*. 229, 271-276.

Somdee T., Peekan A. & Somdee A. 2013. Bacterial degradation of microcystins within a biologically active sand filter. *J. Life Sci. Technol. Vol*. 40, 768-774.

Somdee T., Thunders M., Ruck J., Lys I., Allison M. & Page R. 2013. Degradation of [] MC-LR by a Microcystin Degrading Bacterium Isolated from Lake Rotoiti, New Zealand. *ISRN microbiology*. 2013,

Takenaka S. & Watanabe M. F. 1997. Microcystin LR degradation by *Pseudomonas aeruginosa* alkaline protease. *Chemosphere*. 34, 749-757.

Tamura K., Dudley J., Nei M. & Kumar S. 2007. MEGA4: Molecular Evolutionary Genetics Analysis (MEGA) software version 4.0. *Mol Biol Evol*. 24, 1596-1599.

Tsuji K., Asakawa M., Anzai Y., Sumino T. & Harada K.-i. 2006. Degradation of microcystins using immobilized microorganism isolated in an eutrophic lake. *Chemosphere*. 65, 117-124.

Valeria A. M., Ricardo E. J., Stephan P. & Alberto W. D. 2006. Degradation of Microcystin-RR by *Sphingomonas* sp. CBA4 isolated from San Roque reservoir (Cordoba - Argentina). *Biodegradation*. 17, 447-455.

Yan H., Wang J., Chen J., Wei W., Wang H. & Wang H. 2012. Characterization of the first step involved in enzymatic pathway for microcystin-RR biodegraded by *Sphingopyxis* sp. USTB-05. *Chemosphere*. 87, 12-18.

Yan H., Wang H., Wang J., Yin C., Ma S., Liu X. & Yin X. 2012. Cloning and expression of the first gene for biodegrading microcystin LR by *Sphingopyxis* sp. USTB-05. *Journal of Environmental Sciences*. 24, 1816-1822.

Yang F., Zhou Y., Yin L., Zhu G., Liang G. & Pu Y. 2014. Microcystin-degrading activity of an indigenous bacterial strain *Stenotrophomonas acidaminiphila* MC-LTH2 isolated from Lake Taihu. *PloS one*. 9, e86216.

Yang F., Zhou Y., Sun R., Wei H., Li Y., Yin L. & Pu Y. 2014. Biodegradation of microcystin-LR and-RR by a novel microcystin-degrading bacterium isolated from Lake Taihu. *Biodegradation*. 25, 447-457.

Zhang J., Shi H., Liu A., Cao Z., Hao J. & Gong R. 2015. Identification of a New Microcystin-Degrading Bacterium Isolated from Lake Chaohu, China. *Bulletin of environmental contamination and toxicology*. 94, 661-666.

Zhang M., Pan G. & Yan H. 2010. Microbial biodegradation of microcystin-RR by bacterium *Sphingopyxis* sp. USTB-05. *J of Environ Sci*. 22, 168-175.

Zhang M., Yan H. & Pan G. 2011. Microbial degradation of microcystin-LR by *Ralstonia solanacearum*. *Environl Tech*. 32, 1779-1787.

Zhang X., Hu H.-Y., Hong Y. & Yang J. 2008. Isolation of a Poterioochromonas capable of feeding on *Microcystis aeruginosa* and degrading microcystin-LR. *FEMS Micro Lett*. 288, 241-246.

Zhu X., Shen Y., Chen X., Hu Y. O., Xiang H., Tao J. & Ling Y. 2016. Biodegradation mechanism of microcystin-LR by a novel isolate of *Rhizobium* sp. TH and the evolutionary origin of the *mlrA* gene. *Intl Biodeter & Biodegrad*. 115, 17-25.
